# Speech Task Force and Quality of Life after Surgery in Children with Cleft Lip and Palate: Limitation of Professionals

**DOI:** 10.1055/s-0043-1776738

**Published:** 2024-04-04

**Authors:** Benjamas Prathanee, Panida Thanawirattananit, Phrutthinun Surit, Ratchanee Mitkitti, Kalyanee Makarabhirom

**Affiliations:** 1 Department of Otorhinolaryngology, Faculty of Medicine, Khon Kaen University, Khon Kaen, Thailand; 2 Department of Biochemistry, Faculty of Medical Sciences, Naresuan University, Mueang, Phitsanulok, Thailand; 3 Department of Community Nursing, School of Nursing, Mae Fah Luang University, Chiang Rai, Thailand; 4 Department of Communication Sciences and Disorders, Faculty of Medicine, Ramathibodi Hospital, Mahidol University, Bangkok, Thailand

**Keywords:** speech task force, quality of life, speech therapy, limited resources, lack professional

## Abstract

**Background** Shortage of speech and language therapists results in lack of speech services. The aims of this study were to find the effectiveness of a combination speech therapy model at Level IV: General speech and language pathologist (GSLP) and Level V: Specific speech and language pathologist (SSLP) in reduction of the number of articulation errors and promotion the quality of life (QoL) for children with cleft palate with or without cleft lip (CP ± L).

**Methods** Fifteen children with CP ± L, aged 4 years 1 month to 10 years 9 months (median = 76 months; minimum:maximum = 49:129 months) were enrolled in this study. Pre- and post-assessment included oral peripheral examination; articulation tests via Articulation Screening Test, Thai Universal Parameters of Speech Outcomes for People with Cleft Palate, Hearing Evaluation, The World Health Organization Quality of Life Brief_Thai (WHOQOL-BRIEF-THAI) version questionnaire for QoL were performed. Speech therapy included a 3-day intensive speech camp by SSLP, five 30-minute speech therapy sessions by a GSLP, and five 1-day follow-up speech camps by SSLP that provided four 45-minute speech therapy sessions for each child.

**Results** Post-articulation revealed statistically significant reduction of the numbers of articulation errors at word, sentence, and screening levels (median difference [MD] = 3, 95% confidence interval [CI] = 2–5; MD = 6, 95% CI = 4.5–8; MD = 2.25, 95% CI = 1.5–3, respectively) and improvement of QoL.

**Conclusion** A speech task force consisting of a combination of Level IV: GSLP and Level V: SSLP could significantly reduce the number of articulation errors and promote QoL.

## Introduction

The most common speech disorders in cleft palate with or without cleft lip (CP ± L) after surgery are deviant consonant production, hypernasality, and audible or nonaudible nasal emission. Prevalence of deviant consonant production in children with CP ± L had been shown to be 34%. 1 Poorer results, particularly articulation disorders were in the range of 71.18 to 83.8% after primary palatoplasty. 2 3 4

Speech defects, particularly articulation disorders, result in negative daily life communications and social relationships. Articulation disorders require a prolonged period of speech intervention in children with CP ± L. 2 3 4 5 Speech therapy is a critical concern during the preschool period to prepare children to be able to participate in society and establish social relationships in school. 6 The shortage of speech and language therapists (SLPs) in some developing and underdeveloped countries, especially in low- and middle-income countries (LMICs) is still critical.

A speech task force is a common way to solve these barriers. Many models of speech task forces for children with CP ± L have been implemented in countries such as Mexico, 5 Uganda, 7 Thailand, 8 and the Lao People's Democratic Republic LPDR. 9 A consensus approach for providing speech services for children with CP ± L in India was community-based rehabilitation programs. 10 Concurrent sessions from the following: Task force programs from the 14 ^th^ International Cleft Congress of Cleft lip, Palate and Related Craniofacial Anomalies, Edinberg, 2022—a group of SLPs or task force members in a total of 14 participants from 13 countries across 5 continents, had summaries of categorizing different services. The speech task force that provided speech and language therapy for individuals with CP ± L was divided into five levels: Level I: Grassroot level (community workers); Level II: Paraprofessionals or other health care cleft professionals; Level III: Speech assistants; Level IV: General speech and language pathologists (GSLPs); Level V: Specific speech and language pathologists (SSLPs) 11 . Level II: Paraprofessionals or other health care cleft professionals was performed in Thailand 8 and was extended to the LPDR 9 based on their facilities and support systems. All speech task forces resulted in positive outcomes.

CP ± L also has a profound impact on social interactions and quality of life (QoL) of patients and their families. 12 Previous studies indicated that children with CP ± L had a lower QoL than normal peers. 12 13 Children with CP ± L, however, received face teasing from friends 14 and had fewer interactions with both speech and physical signs of interest. 15 They, therefore, had a negative self-perception. 12 15 Preschool aged children generally have rapid development of cognitive skills, socioemotional competence, and interactive behavior and this is a critical time for acceptance by peers, resulting in increased self-perception and personality formation. 6 Promotion of QoL needs for these children is essential.

There are generally many barriers and resources to cleft lip and palate speech services in some countries. 16 17 Thailand is an upper middle economy country where there is a shortage of SLPs and lack of speech services for individuals with CP ± L in some areas. The lower northern area is one area that lacks speech services. There is a GSLP in a provincial hospital in this region. Children with CP ± L who were registered in the Naresuan Cleft and Craniofacial Center, Faculty of Medicine, Naresuan University, which had no SLP. These children did not get speech services because of lack of resources including long-distance access to speech services from their areas in the central part or upper northern parts where speech service is available. Most patients were poor; most parents worked in cities far from hometown and grandparents took care of the children. Family perception and the community had concerns; however, they could not get support in expenses and time to get speech therapy from the nearest speech center in upper north or central Thailand. A speech task force would help to reduce articulation errors.

The aim of this study was to find the effectiveness of a speech therapy model: a combination of Level IV: GSLP and Level V: SSLP in reduction of the number of articulation errors and the effectiveness of promotion of QoL for children with CP ± L in the lower northern area of Thailand.

## Methods

This study was a pre- and post-prospective clinical study. This sample size was calculated based on study of pre- and post-articulation therapy for children with CP ± L. 9 Children with CP ± L, aged 4 to 12 years who were registered for treatment at the Naresuan Cleft and Craniofacial Center, Faculty of Medicine, Naresuan University, and children who had no previous speech therapy were included. They were preschool and middle school children who were in the appropriate period of preparation to be part of the peer society and social relationships in school. Inclusion criteria were children with CP ± L who had already been treated and with exclusion criteria for children with CP ± L and with moderate hearing loss (both ears) or congenital defects or global delayed development (e.g., mental retardment, autism, cerebral palsy, etc.) with more than two articulation errors (not including /r/—the most common error in Thai language and there is no exact age for Thai children with these errors). Twenty children with CP ± L were registered in this study. Five of them were excluded because they had less than two or no articulation defects after preperceptual assessment. Fifteen children with CP ± L were enrolled in the study. All of them were prepared as follows:

Pre- and post-perceptual assessments were performed.

1. Oral peripheral examination
2. Short conversations for eliciting understandability and acceptability (e.g., “What is your name?,” “What is your school's name?,” “Tell me how you travelled to speech camp?,” “What did you play or do yesterday and what did you do today?,” “What kind of food do you like?,” etc.) This made children familiar with how to build a social relationship.
3. The Articulation Screening Test on how to build simple connected speech 18 was composed of four connected sentences which covered all Thai consonants with pictures and was performed for eliciting speech outcomes at the screening level.
4. Thai Universal Parameters of Speech Outcomes for People with Cleft Palate 19 was used for pre- and post-perceptual assessment to elicit speech outcomes. This was established based on Thai phonetics and speech sampling guidelines for universal parameters for reporting speech outcomes in individuals with cleft palate. 20 It was composed of all Thai tones, vowels, consonants, and seven typical patterns of speech characteristics in patients with cleft palate, particularly consonant production errors. Speech samples had drawn pictures with texts in 41 words and 36 sentences. Naming pictures was performed or imitations were provided in case of children who could not read or name the pictures.
5. Hearing test: audiogram with audiometer (audiometer: Madsen Voyager: vr522) was performed by a qualified audiologist.
6. The WHOQOL-BRIEF-THAI version questionnaire was used for investigation of the pre- and post-perceptual impact of QoL. This questionnaire explored caregivers' feeling about the patient's QoL, health, or other areas of their lives. The caregivers who could read could fill in questionnaire by themselves. If they were illiterate, researchers interviewed the caregiver and filled in the questionnaire. The WHOQOL-BRIEF-THAI version questionnaire consisted of 4 major parts and 26 items, including Physical health (7 items of the questions No. 2–4, 10, 11, 12, and 24), Psychological health (6 items of the question No. 5–9 and 23), Social relationship (3 items of the question No. 13, 14, and 25), Satisfaction with Environment (8 items of the question No. 15–22), and 2-item overall QoL (question No. 1 and 26). QoL scores would range from 1 to 5 on the Likert scale and the overall QoL possible would range in the sum scores of 25 to 112. The score was categorized into three levels: poor QoL (sum score ranged from 9 to 21), fair QoL (sum score ranged from 22 to 33), and good QoL (sum score ranged from 34 to 45). 21
7. The UTAH language test was used as a language screening test for language assessment. 22 This test is a language screening evaluation that is composed of both expressive and receptive languages based on the child's age. If child could do or pass all items of test, language skill might be normal or with a mild delayed speech and language development. If child could not pass any item, language skill was delayed.

The main speech outcome was an articulation error from perceptual assessment. In-person pre-and post-articulation tests were consensus between two investigators (first and fifth). If there was not a consensus, a retest was performed until there was consensus.

**Fig. 1:**
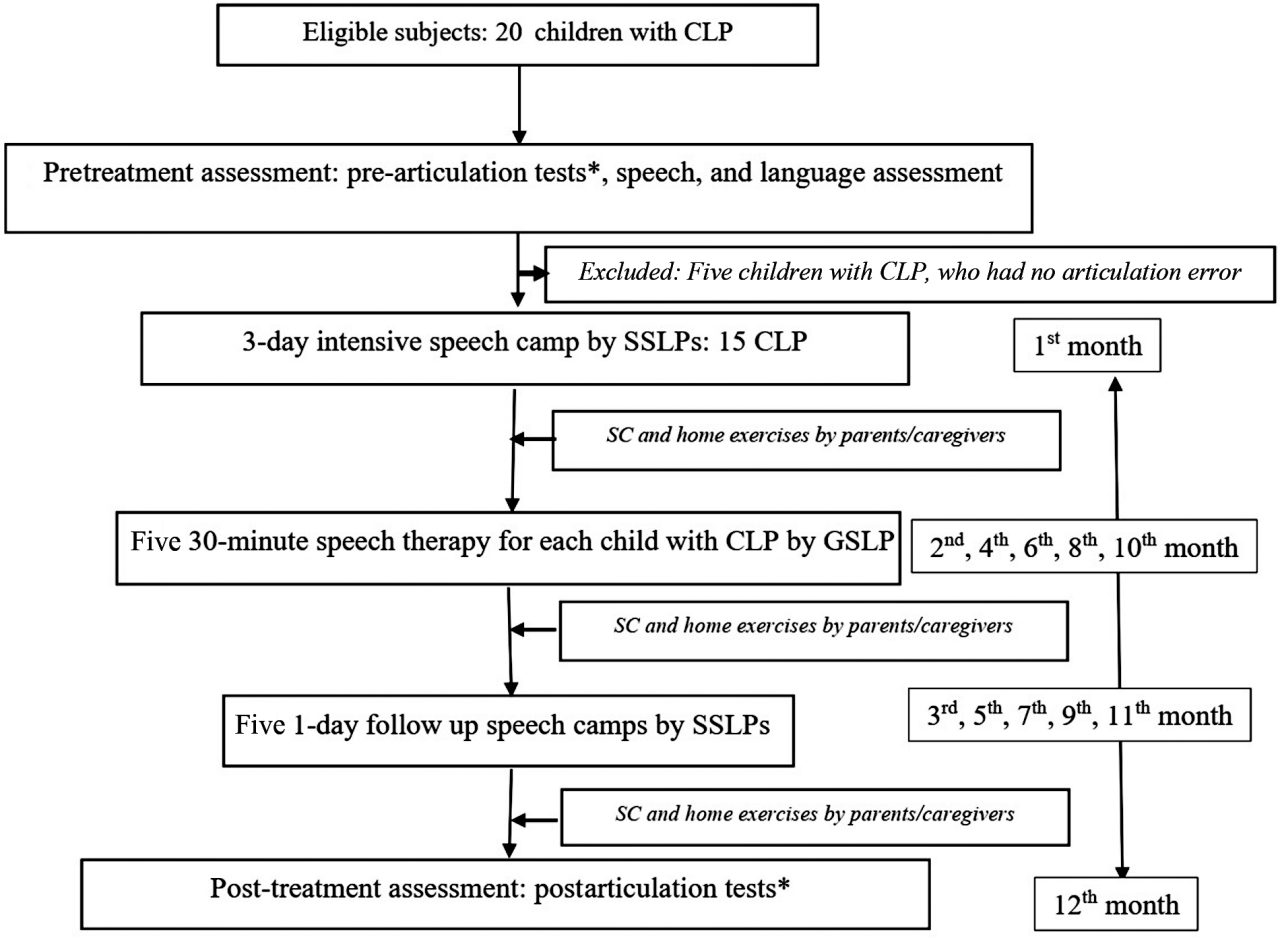
Design outline. *Thai Universal Parameters of Speech Outcomes for People with Cleft Palate and Articulation Screening test. CLP, cleft lip and palate; GSLP, general speech and language pathologist; SC, speech correction; SSLP, specific speech and language pathologist.

The speech task force was speech therapy model: a combination of Level V: GSLP and Level IV: SSLP. The design outline is displayed in Fig. 1 .

The process of speech task force was composed of (1) a 3-day intensive speech camp conducted by three SSLPs and a GSLP at Naresuan Cleft and Craniofacial Center. Both SSLPs and GSLP used the protocol of the guide book for speech correction. 18 SSLPs demonstrated and taught speech correction for children with CP ± L to GSLP and caregivers in a case by case method based on individual articulation errors and approaches for correction that each child successfully learned. GSLP and caregivers practiced the assigned home exercises with SSLP supervision. The record book of speech therapy 23 and exercise of articulatory correction for children with CP ± L 24 methods were given and introduced to GSLP and caregivers. pre-perceptual assessment and four 45-minute speech therapy sessions/day/child were provided for 15 children with CP ± L by three SSLPs and a GSLP with SSLPs under supervision (first month). Four stations of speech therapy with individual and group practice and a station for promotion of QoL were provided for children with CP ± L and caregivers. Rotation of stations every 45 minutes was run by a research assistant with a 15-minute break for a snack; (2) five 30-minute follow-up speech camps by a GSLP provided a 30-minute speech therapy for each child every 2 months (second, fourth, sixth, eighth, and tenth month); (3) five 1-day follow-up speech camps similar to arrangements in the 3-day intensive speech camps, four 45-minute speech therapy sessions/day/child were provided by three SSLPs and a GSLP with supervision by SSLPs for 15 children with a CP ± L every 2 months (third, fifth, seventh, ninth, and eleventh month). SLPs summarized home exercise and caregivers filled in home practice in a calendar of the record book. Monitoring home program and practice was done via individual record books of speech therapy. 23

Caregivers practiced home program at home for four to five 20-minute sessions/week. Post-perceptual assessment was done at the final session (twelfth month). This project supported all family expenses.

Phonological, traditional approaches, and specific strategies in speech correction that are based on protocols of the guide book for speech correction were individually introduced and demonstrated for caregivers by SSLPs based on teaching services. The first priority for training of speech therapy was started at isolated sounds, then the syllable level for every sound moved to the next steps: 1-syllable single word, 2-syllable word/phrase, 3-syllable word/phrase, sentence, reading, and storytelling with a focus on target sounds. Home exercises were assigned to caregivers based on exercises for articulatory correction for children with CP ± L. 24

For QoL, after questionnaire assessment, findings were summarized and the intervention planned. The root causes of the problems were identified and the goals for development stimulation and problem-based solving between team and children with CP ± L's family were established. Intervention and consulting related to all domains including physical health, psychological health, social, satisfaction with environment, and individual weak points were provided throughout 3-day intensive speech camps and five 1-day follow-up speech camps. Researchers gave promotion for QoL in all aspects including the home program. The WHOQOL-BRIEF-THAI version questionnaire was used for postevaluation of QoL.

Descriptive statistical analysis was used for determining children's characteristics, The main outcomes of this study were articulation errors during the pre- and poat-articulation tests. Articulation was scored as 0, correct or normal; 1, incorrect or error. Data were entered into Excel 2013 (Microsoft Corp., Redmond, WA). Wilcoxon signed-rank test was used to determine the effectiveness of speech task force for children with CP ± L by comparing the number of pre- and postarticulation errors as well as pre- and post-scored of the WHOQOL-BRIEF-THAI version questionnaire. Significance of the *p* -values was indicated as the median differences (MDs).

## Results

Characteristics are summarized in Table 1 . Fifteen children with CP ± L (male:female = 6:9), median age was 76 months (minimum:maximum= 49:129 months), had complete participation in the speech test group. UTAH assessment revealed all children had normal language development with exception of NU03 who had delayed speech and language development. Travelling from participants' homes to Naresuan Cleft and Craniofacial Center for getting intensive and 1-day follow-up speech camps was approximately 57 to 132 km and an average time from home to center around was 2 to 4 hours, depending on traffic and local road situations. Most of them had normal hearing, three children (NU09 and NU19) had only one-ear mild hearing loss, one child had hearing in both ears (NU10) above normal (mild hearing loss) that did not interfere with the outcomes. A child with CP ± L had mild hearing level in right ear (40 dB) and moderate hearing loss in left ear (45 dB; NU14). All children with hearing abnormalities were referred to otorhinolaryngologist for further treatment and then for follow-up, particularly, after every visit of speech follow-up (once a month).

**Table 1:** General characteristics of children with cleft lip and palate

Variables	Number/others	Percentage/SD
**Gender**		
•Female	6	40
•Male	9	60
**Age (months)**		
•Median	76	–
•Min:Max	49:129	–
** Genetics (CLP) a**		
•Yes	1	6.67
•No	14	94.00
**Native language**		
•Thai	4	26.67
•Northern	11	73.33
**Age of lip repair (months)**	Ⴟ = 4	SD = 0.20
**Age of first palatoplasty (months)**	Ⴟ = 12.14	SD = 0.54
**Diagnosis**		
•Left unilateral CLP	5	33.33
•Right unilateral CLP	4	26.67
•Bilateral CLP	5	33.33
•Cleft palate	1	6.67
**Hearing**		
•Normal hearing both ears	10	66.67
•Unilateral mild hearing loss	3	20.00
•Bilateral mild hearing loss	1	6.67
•Unilateral mild hearing loss and moderate hearing loss	1	6.67

Abbreviations: CLP, cleft lip and palate; SD, standard deviation; Ⴟ, mean.

a There was a family member (uncle) with cleft lip and palate.

Pre- and post-perceptual assessment at word and sentence levels, and Articulation Screening Test results are provided in Table 2 . Results display individual articulation errors before and after speech task force work. All children with CP ± L had significant improvement in articulation errors.

**Table 2:** Number of articulation errors

ID	Sentence (sounds)			Word (sounds)			Screening (sounds)		
	Pre	Post	Reduction	Pre	Post	Reduction	Pre	Post	Reduction
NU01	8	5	3	3	3	0	3	1	2
NU03	11	4	7	8	5	3	5	2	3
NU04	13	4	9	13	6	6	6	2	4
NU05	15	5	10	8	5	3	7	4	3
NU06	4	0	4	3	0	3	1	0	1
NU09	22	18	4	21	13	3	14	7	7
NU10	19	14	5	13	6	7	12	11	3
NU11	10	6	4	8	5	3	7	3	4
NU13	6	0	6	0	0	0	2	0	2
NU14	8	2	6	6	1	5	4	1	3
NU15	8	0	8	4	0	4	1	0	1
NU16	7	1	6	2	1	1	3	1	2
NU17	4	1	3	3	0	3	2	1	1
NU18	18	6	12	7	4	3	6	5	1
NU19	8	1	7	2	1	1	3	1	2

Abbreviations: dB, decibel; Pre, prearticulation test; Post, postarticulation test.

The most common types of compensatory misarticulations were glottal, velar, and pharyngeal, and mid-dorsum palatal substitutions. Normal distribution of articulation errors was determined by the Shapiro–Wilk W test and the Wilcoxon sign-rank test was used for determining MD between number of pre- and post-articulation errors ( Table 3 ).

**Table 3:** Comparison of median difference between pre- and postarticulation errors

Level ( *n* = 15)	Median (min:max)		Median difference	95% CI	*p* -Value
	Pre	Post			
Sentence	8 (4:22)	4(0:18)	6	4.5, 8	<0.001
Word	6 (0: 21)	3 (0:13)	3	2, 5	<0.001
Screening	4 (1:14)	1 (0:11)	2.25	1.5, 3	<0.001

Abbreviations: CI, confidence interval; max, maximum; min, minimum.

Comparison of MDs between pre- and post-articulation errors revealed that there were statistically significant reductions of the numbers of articulation errors at all levels: word, sentence, and screening. Subanalysis without NU14 (who had mild hearing loss in right and moderate hearing loss in left ear) outcomes was performed. Results indicated that there was a statistically significant reduction of the numbers of articulation errors at all levels: sentence, word, and screening (MD = 6; 95% confidence interval [CI] = 4.5–8; MD = 3; 95% CI = 1.5–5; MD = 2, 95% CI = 1.5–2.5, respectively).

Pre- and post-QoL scores from the WHOQOL-BRIEF-THAI version are compared and presented in Table 4 . Scores of QoL revealed significant improvement in all domains: physical health, psychological health, social relationship, satisfaction with environment, and overall QoL.

**Table 4:** Comparison of median difference between pretest and post-test of quality of life

Number = 15	Number of items	Possible score	Median (min:max)		Median difference	95% CI	*p* -Value
			Pre-QoL (min:max)	Post-QoL (min:max)			
Physical health	7	7–35	16 (12:32)	27 (21:35)	11	13.12–17.25	<0.001
Psychological health	6	6–30	13 (10:21)	25 (16:30)	12	9.02–16.12	<0.001
Social relationship	3	1–15	6 (4:12)	12 (10:15)	6	4.5–11.32	0.004
Satisfaction with environment	8	8–40	24 (18:32)	36 (30:40)	7	6.23–16.11	<0.001

Abbreviations: CI, confidence interval; max, maximum; min, minimum; Post-QoL, post-test of quality of life; Pre-QoL, pretest of quality of life; QoL, quality of life.

## Discussion

Some children used northern Thai as the native language in their families. They used Thai as the official language in school and community ( Table 1 ). Thai was used mainly for communication in the project. Children and families had no problems in communication. The same intervention was used for participants.

Based on Naresuan Cleft and Craniofacial Center's protocol, children with CP ± L received palatoplasty at around 1 year with the technic of two-flap palatoplasty with intravelar veloplasty and were investigated on ear, nose, throat, hearing evaluation, and treatment every 3 months since birth. Hearing evaluation was performed on the first day of starting the speech camp to ensure the current hearing abilities. Children who had hearing loss were referred to the ENT clinic for proper management. A child with CP ± L who had right ear hearing = 40 dB and left ear of 45 dB (NU14) was also found to have conductive hearing loss in both ears, showed hearing improvement to be normal in the right ear and mild hearing loss in the left ear (right ear = 25 dB; left ear = 28 dB) after treatment.

Comparison of MDs between pre- and post-articulation errors revealed that there was a statistically significant reduction of the numbers of articulation errors at all levels: sentence, word, and screening (MD = 3, 95% CI = 2–5; MD = 6, 95% CI = 4.5–8; MD = 2.25, 95% CI = 1.5–3, respectively). This indicated that the speech task force (combination of speech therapy model: a combination of Level IV: GSLP and V:SSLP) resulted in positive outcomes and can be used as a model for solving lack of speech services in any area where there had been very few or a limitation of professional or resources. Similar results were found in previous studies that used different models and strategies to enhance speech therapy in children with CP ± L in many LMICs. 5 7 8 9 25 26 27 28 29 Regarding hearing, development of language and psychosocial skills are significantly influenced by moderate hearing loss (hearing level = 41–60 dB) that generally may affect children who then fall behind in language skills compared with their normal hearing peers, 30 where average conversational speech loudness is 40 to 60 dB. 31 Most children in this study had normal hearing level in an ear and had normal speech and language development or with a minimal effect on outcomes. This agreed with the UTAH assessment which revealed that all children had normal language development with exception of NU03. For NU14 who had mild hearing loss in right ear and moderate hearing loss in left ear (both ears had conductive hearing loss), post-articulation numbers significantly decreased. This was supported by the previous studies that found conductive hearing loss may not be a substantial risk factor for later speech and language development or academic achievement, 32 and no correlations between speech, language, and auditory function testing measures and pure tone averages thresholds. 33 This subject hearing improved from treatment during speech task force to 25 dB in the right ear and 28 dB in the left ear. Subanalysis without NU14 outcomes was a statistically significant reduction in the numbers of articulation errors at all levels: sentence, word, and screening (MD = 6, 95% CI = 4.5–8; MD = 3, 95% CI = 1.5–5; MD = 2, 95% CI = 1.5–2.5, respectively).

The numbers of intensive speech therapy by the task force were 21 45-minute sessions by SSLPs and 5 30-minute sessions by a GSLP within a year. The speech task force expected the average sessions for home practice by caregivers or parents to be four to five 20-minute sessions/week. Some caregivers sometimes did extra sessions of home practice to be more than or equal to one session every day (NU04, NU05, NU13, NU14, and NU17) while some could not consistently do home practice (NU11: one to two sessions a week). Researchers and teams encouraged and facilitated them to do the best they could. They tried to do more as they could but the number of home practice sessions did not reach the criteria that the project expected because they had limitation situations, for instance, a child with CP ± L lived with single father and grandmother, the child lived with grandmother who had illiteracy problems and could not train speech exercise at home while the father was a truck driver who needed going out and stayed overnight in another district to work. The father came back home and practiced home exercise only once a week. Neighbors or relatives inconsistently helped to practice exercise. Another child who lived with grandmother and was abused by relatives needed to be referred to a psychiatrist, psychologist, and social worker. In observation of Table 2 , data presented significant reductions of articulation errors in the group of children who received home practice to be more than or equal to one session (NU04, NU05, NU13, NU14, and NU17) and there was also improvement in a child who had fewer sessions of home practice (NU11). The formal data for significant comparison of the outcomes between two groups were inadequate for conclusion. It might be interesting to explore difference of the speech outcomes in further study. For the different ages and severity of CP ± L participants in this study, the outcomes might be affected. Some children who still had hypernasality or velopharyngeal insufficiency were referred to surgery at the end of project.

The results of the analysis of QoL of children with CP ± L and their families found that in the overall picture of the project, there was a good trend in every aspect from pre- and post-tests of their QoL: physical health (MD = 11, 95% CI = 13.12–17.25), the psychological health (MD = 12; 95% CI = 9.02–16.12), the social relationship (MD = 6; 95% CI = 4.50–11.32), the satisfaction with environment (MD = 7; 95% CI = 6.23–16.11, *p* -value <0.01), and overall QoL (MD = 32; 95% CI = 27.23–42.36, *p* -value <0.01). Speech force should focus on both speech therapy and related factors including psychosocial problems and poor economic status from long-term treatment. This psychological stress might be reduced if addressed by specialist clinical psychologists in cleft-treating centers. 34 These factors were also supported by the speech task force.

Regarding satisfaction, caregivers gave a positive impression of speech task forces as “very good activities,” “my child clearly speaks,” “my child has more confidence to speak,” and “staff are so kind.” There were no negative expression.

This model, a combination of GSLP and SSLP, had results supported to findings of other speech therapy models including at the speech task force Level II 8 9 and Level IV 5 25 35 36 37 in significant reduction in the number of articulation errors. This appears to be a way to solving lack of speech services for children with CP ± L from limitation of professionals. For further management of situations where there is a lack of professionals and speech therapy approach would be sustainable, GSLP in the north that planned to continue education for the SSLP and Naresuan Cleft and Craniofacial Center tried to find out an SLP to be staff.

Limitations of the study, sample size might be small and hearing problems might make the samples' baseline characteristics not equivalent, sessions of home exercise practice in each child might lack consistency that were beyond the scope of the study's control even though there was a system to encourage and monitor caregivers to practice exercises at home via book records and suggestions. This might affect the outcomes of this study.

### Conclusion

The speech task force using the speech therapy model: a combination of GSLP and SSLP significantly reduced the number of articulation errors and promoted QoL. This technique should be compared with other speech therapy models in similar patient populations in the future.
